# The Effect of Wheat Bran on the Chemical Composition, Texture, and Oxidative Stability of Beef Meatballs

**DOI:** 10.3390/foods15101687

**Published:** 2026-05-12

**Authors:** Daniela Ianiţchi, Liliana Aurelia Bădulescu, Paula Poşan, Elena Narcisa Pogurschi, Monica Paula Marin, Violeta Alexandra Ion, Carmen Gabriela Constantin, Aurora Dobrin, Livia Patraşcu, Camelia Hodoşan, Marius Laurian Maftei

**Affiliations:** 1 Faculty of Animal Production Engineering and Management, University of Agronomic Sciences and Veterinary Medicine of Bucharest, 59 Marasti Blvd, 011464 Bucharest, Romania; daniela.ianitchi@usamv.ro (D.I.); elena.pogurschi@usamv.ro (E.N.P.); monica.marin@usamv.ro (M.P.M.); camelia.hodosan@igpa.usamv.ro (C.H.); marius.maftei@usamv.ro (M.L.M.); 2 Research Center for Studies of Food Quality and Agricultural Products, University of Agronomic Sciences and Veterinary Medicine of Bucharest, 59 Marasti Blvd, 011464 Bucharest, Romania; liliana.badulescu@qlab.usamv.ro (L.A.B.); violeta.ion@qlab.usamv.ro (V.A.I.); carmen.constantin@qlab.usamv.ro (C.G.C.); aurora.dobrin@qlab.usamv.ro (A.D.); 3 Cross-Border Faculty, Dunarea de Jos University of Galati, 111 Domneasca Str, 800201 Galati, Romania; livia.patrascu@ugal.ro

**Keywords:** beef, wheat bran, fatty acids, amino acids, minerals, antioxidant activity

## Abstract

The use of wheat bran (WB) to obtain functional meat products is a concept to be studied, due to sustainability issues and the nutritional advantages related to the decrease in caloric value, and the intake of phytonutrients, which can limit the incidence of diseases such as obesity, diabetes, or colon cancer. Beef meatballs with added wheat bran (5, 10, and 15%) were analyzed in terms of their physico-chemical parameters: pH, proximal composition, fiber content, caloric value and texture, antioxidant activity, fatty acids, amino acids, mineral content, and peroxide value. The fiber concentration in the meatballs increased proportionally with the WB addition. Although the antioxidant activity of the meatballs with WB was higher at the time of sample production, their stability proved to be weaker over time. The addition of wheat bran resulted in an overall decrease in amino acid concentrations and an increase in polyunsaturated fatty acid concentrations from 9.32 g/100 g to 14.36 g/100 g. For Mg, Ca, Mn, Cu, and Cr, there were significant increases between groups (*p* < 0.001), while Na and Zn decreased with the addition of wheat bran. The peroxide value (PV) and free acidity (FFA) decreased with the increase in the proportion of added fiber. The increase in the percentage of wheat bran also generated significant reductions (*p* < 0.001) for all textural parameters investigated. Including wheat bran in meatballs can present technical challenges related to composition cohesion and product shaping, as well as sensory acceptance. At the industrial scale, problems may arise related to the processing equipment, and the shaping of meatballs becoming difficult with the increased wheat bran addition.

## 1. Introduction

Meat consumption contributes to a balanced diet, meat being a rich source of essential nutrients such as essential amino acids, vitamins, especially from group B, minerals, and lipids [1]. Worldwide, meat consumption is a highly debated topic, with concerns about its impact on human health, the environment, and animal welfare [2]. The exploitation of meat analogues such as fibers including orange, wheat, bamboo, carrot, dragon fruit peel powder, apple, oat, pea, inulin, plant gums and hydrocolloids [3], soy protein derivatives, lupin, and chickpea [4] for the development of new meat products is promising and may offer multiple opportunities.

The use of various dietary fibers, limited in terms of energy, has the advantage of reducing the caloric value of the finished products [5], accelerating intestinal transit, facilitating the elimination of carcinogenic substances, positively influencing the gut microbiome [6], decreasing the concentration of cholesterol and serum lipids [7]. Moreover, flours obtained from cereals, roots, and legumes present various other benefits, in addition to the fiber content, such as antioxidant activity [8], and a high content in polyunsaturated fatty acids, vitamins [9], and phytosterols [10], substances that can increase the nutritional value of the products in which they are incorporated. These fibers are important for their health benefits, offering protection against diseases associated with an unhealthy lifestyle [11]. The recommended daily intake of dietary fiber is 28–36 g, of which 70–80% should be insoluble fiber. Insoluble dietary fiber is associated with intestinal regulation, while soluble fiber has implications in lowering cholesterol levels and intestinal glucose absorption [12].

From a technological perspective, the introduction of fibers into meat products is an increasing trend due to their numerous functional properties such as water retention, the emulsification capacity, the ability to reduce losses during technological processes, or texture improvement [3,13,14].

Wheat bran is a good source of dietary fiber and is frequently used in the management of conditions such as constipation and diabetes, as well as for reducing carcinogenic compounds in the body [10]. Therefore, the existence of numerous studies aimed at exploiting these fibers for the development of new meat products is justified. Wheat bran has been used not only as a fat substitute in the production of beef sausages, with positive effects on improving the water retention capacity and plasticity, and reducing cooking losses [5], but also in the production of beef meatballs with positive effects on reducing cooking losses, the fat content, and saturated fatty acids [15]. Beef burgers obtained with the addition of different cereals, including wheat, demonstrated improved oxidative stability, without sensory impairment (according to the study by Ramadan) [8]. Adding cereal bran (wheat, corn, rye, and oats) to beef patties resulted in a reduced fat content, cooking weight loss, and increased fiber content [16]. Developing a new functional meat product, a cooked sausage enriched with wheat bran, linseed oil, and vitamin premixes, resulted in increased vitamin B2, vitamin E, and omega-3 fatty acid content, as well as a significant improvement in texture [17]. The use of plant derivatives as a coating base for meat products directly influenced the behavior of the product during heat treatment, oil absorption, and moisture retention [18]. However, while an improved oxidative stability and increased fiber concentration has been observed, the use of modified wheat bran fibers as fat substitutes in beef sausages has also been associated with a deterioration in texture [19].

The incorporation of plant fibers is appropriate not only from a nutritional perspective, but also from an economic and environmental perspective, as there is increasing consumer concern for sustainable and eco-friendly food options [2]. Dietary fibers are generally secondary agricultural products, which are relatively inexpensive, and their incorporation as natural antioxidants in meat products reduces production costs [20]. On the other hand, the growing demand for “clean label” products has increased interest in replacing synthetic additives with natural, plant-derived antioxidants [21].

Although numerous studies have investigated the use of various plant fibers in the development of functional meat products, limited information is available regarding the combined effect of wheat bran incorporation on the chemical composition, oxidative stability, and degradation behavior of beef patties during storage. Beef patties represent a widely consumed meat product and a suitable model system for evaluating functional ingredient incorporation due to their susceptibility to lipid oxidation and structural modifications during processing and storage.

This study provides an integrated assessment of the nutritional composition (the protein, lipid, and mineral profile), and oxidative and degradation behavior in wheat-bran-enriched beef patties, in a formula analogous to those used in industrial practice.

## 2. Materials and Methods

### 2.1. Raw Materials

The study was conducted on formulations consisting of beef, salt, water, spices, and wheat bran added in amounts of 5, 10, and 15% according to Table 1. The raw material, represented by beef Gluteus muscle, purchased from SC Intern SRL, Romania, at 24 h after slaughter, presented sensory properties specific to fresh refrigerated meats: red color, slightly moist on the surface, with shiny, elastic, and hard connective tissues, elastic consistency, and pleasant, characteristic odor. The chemical composition of the beef muscle used was 72.67% water, 20.95% protein, 5.59% lipid, and 1.13% ash.

Wheat bran, purchased from Driedfruits Romania, was presented as a coarse powder with the following chemical composition, according to the specifications on the label: 16% protein, 4.7% lipid, 17.5% carbohydrates, and 45.4% fiber. The spices, represented by mixed peppercorns (50%) and dehydrated garlic flakes (50%), are produced by Driedfruits, Romania. According to the specifications on the label, the chemical composition of the mixed peppercorns was 2% fat, 58.6% carbohydrates, 26% fiber, and 10% protein, and the composition of the dried garlic flakes was 0.76% fat, 73% carbohydrates, 9% fiber, and 17% protein.

**Figure 1:**
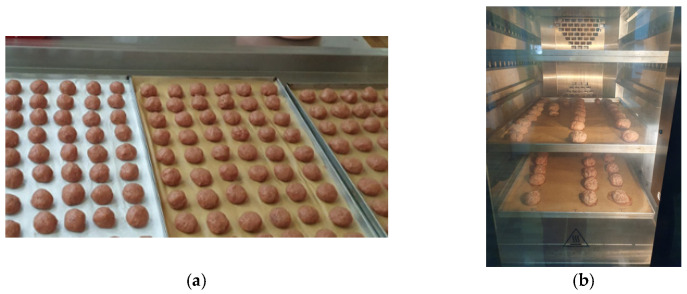
Images during the obtaining process of beef meatballs with wheat bran: (**a**) raw meatballs; and (**b**) meatballs during thermal processing.

The products were obtained by mincing the meat at 3000 rpm for 15 s, with Robot Blixer 3 (Robot Coupe, Vincennes, France), to which the previously hydrated wheat bran and the rest of the ingredients were added, followed by mixing, forming meatballs with a diameter of 5 cm, and cooking at 100 °C, for 30 min (Oven Zanolli Rotor Wind 1E, Zanolli Forni Srl, Verona, Italy), without adding fat (Figure 1). All samples were analyzed after cooling when samples reached room temperature, and, then, for stability assessments, pH, antioxidant properties, and peroxide value were determined after 3, 6, and 9 days of storage at 0–4 °C.

### 2.2. Chemical Proximate Composition and pH

The methods used to determine proximate chemical composition were as follows: AOAC 39.1.02 method was used to determine moisture content by air drying in an SLW 53 oven (Pol-Eko, Wodzisław Sl, Poland). The Kjeldahl method AOAC 39.1.15 with a nitrogen-to-protein conversion factor of 6.25 was used to determine crude protein. The fat content was determined by AOAC 39.1.08 method with a Soxhlet extractor (Gerhardt GmbH & Co., KG, Königswinter, Germany), and AOAC 39.1.09 method was applied for Ash content determination through calcination in an LAC s.r.o. oven (Židlochovice, Czech Republic). pH value of samples was measured over a nine-day storage period, using a pH-meter (ETI 8100 pH Meter, Electronic Temperature Instruments Ltd., Worthing, UK) equipped with interchangeable electrode [22].

### 2.3. Fiber Content

To identify and quantify cellulose and lignin in meatballs samples, acid detergent fiber (ADF) and acid detergent lignin (ADL) analyses were performed according to the Van Soest method [23], using a FIWE 6 Fiber Analyzer (VELP Scientifica Srl, Usmate Velate, Province of Monza e Brianza, Italy). Briefly, 1 g of sample was treated for approximately 1 h with an acid detergent solution to remove starch, pectin, hemicellulose, fats, oils, proteins, free sugars, and soluble minerals. The remaining residue was dried at 100 °C for 8 h and weighed. This residue contained cellulose and lignin. Cellulose solubilization was done by incubating the residue with 72% sulfuric acid for 3 h, followed by drying at 100 °C for 8 h. The final residue was represented by lignin. ADF and ADL were calculated using Equation (1), and cellulose was calculated from the difference:

(1) $$ADF/ADL\;\left(\%\right)=\frac{M_{1}\;-M_{2}}{M_{0}}\times 100$$

where *M*_0_—weight of the sample taken for analysis, g; *M*_1_—mass of the crucible with residue, g; and *M*_2_—mass of the crucible, g.

### 2.4. Mineral Content

To 0.100 g of sample weighed on an analytical balance (Partner AS 220 model, Radwag Balances and Scales, Radom, Poland), the following reagents were added: 8 mL of Suprapure 65% HNO_3_ (Merck, Darmstadt, Germany) and 2 mL of Suprapure 30% H_2_O_2_ (Merck). The samples were digested using an ETHOS UP microwave digestion system (Milestone Srl, Sorisole, Italy). After digestion, the samples were brought to a final volume of 50 mL with ultrapure water (Merck Millipore Direct 8/16) and subjected to elemental analysis using an Agilent Technologies model 7700 (Agilent Technologies, Inc. Headquarters, Santa Clara, CA, USA) inductively coupled plasma mass spectrometer (ICP-MS) [24].

### 2.5. Fatty Acid Content

Fatty acids (FA) were determined by gas-chromatography. The extracted fat from the sample was subjected to methylation to convert the fatty acids from triglycerides into methyl esters (FAMEs), followed by extraction in hexane, separation in capillary columns, and identification and quantitative determination using standard chromatograms [25]. A Perkin Elmer Clarus 500 chromatograph (Waltham, MA, USA) was used. The results were expressed as g/100 g fatty acids methyl esters (FAMEs). The sum of PUFA/SFA/UFA fatty acids was calculated.

### 2.6. Amino Acid Content

Amino acid determination was performed at ICA Research & Development, Bucharest, Romania, by ion exchange chromatography with post-column ninhydrin derivatization (65), after acid hydrolysis (HCl 6N, at 110 °C, for 24 h to convert cysteine to cysteic acid and methionine to methionine sulfone) and HPLC with fluorescence detector (FLD) for specific amino acids, after hydrolysis with sodium hydroxide (NaOH) at 110 °C for 22 h [26].

### 2.7. Antioxidant Activity Determination

The DPPH radical scavenging activity of meatball extracts was determined over a nine-day storage period in order to determine the effect of wheat bran supplementation on oxidative stability of samples. A quantity of 2 g of fresh sample was weighed and mixed with 40 mL of 50% ethanolic solution. The mixture was homogenized using a T25 ULTRATURAX^®^ (IKA-Werke GmbH & Co., KG, Staufen im Breisgau, Germany) at 9000 rpm for 1 min. Extracts were placed in a SONOREX ultrasonic bath (BANDELIN electronic GmbH & Co., KG, Berlin, Germany) at 50 °C for 30 min, then centrifuged for 10 min at 10,000 rpm [27]. Antioxidant activity was determined using the DPPH assay, based on the stable free radical 2,2 diphenyl 1 picrylhydrazyl (DPPH). For each determination, 1 mL of extract was mixed with 2 mL of 0.1 mM DPPH solution in ethanol. The mixture was kept in the dark for 30 min, after which absorbance was measured at 515 nm using a Specord 210 Plus spectrophotometer (Analytik Jena GmbH+Co., KG, Jena, Germany). The blank sample was prepared by replacing sample extract with 50% ethanolic solution. DPPH was calculated using Formula (2):

(2) $$Radical\;scavenging\;activity\;\left(\%\right)=\frac{ABlank\;-Asample}{ABlank}\times 100$$

where *ABlank*—absorbance of the blank sample; and *Asample*—absorbance of the sample extract.

### 2.8. Peroxide Value Determination

The peroxide value was determined by the iodometric method [28] during a nine-day storage period. The previously extracted fat was dissolved in chloroform and acetic acid, treated with potassium iodide, incubated for 5 min, and titrated with sodium thiosulfate. The results were expressed in O_2_ meq/kg fat. Peroxide value (PV) was calculated using Formula (3):

(3) $$Peroxide\;Value\;\left(meqO_{2}/kg\right)=\frac{\left(V1-V2\right)Nx\times \;1000}{M}$$

where *V*1—volume of sodium thiosulfate used for sample, ml; *V*2—volume of sodium thiosulfate used for blank, ml; *N*—sodium thiosulfate solution normality; and *M*—weight of the sample, g.

### 2.9. Free Fatty Acid Determination

The determination of free fatty acids (FFAs) was performed by titrimetric analysis: 5 g of sample homogenized with 50 mL neutralized ethanol-diethyl ether (1:1) solution was mixed with a few drops of phenolphthalein indicator, and the filtered extract was titrated with 0.10 M NaOH until a persistent pink color was reached for a few seconds. FFA, expressed in % oleic acid, was calculated based on the volume of NaOH used in the titration [29]. Formula (4) was used to calculate FFA:

(4) $$FFA\;\left(\%\;oleic\;acid\right)=\frac{V\times M\times 28.2}{m}$$

where *V*—volume of NaOH used in the titration, ml; *M*—molarity of the NaOH solution; and *m*—weight of the sample, g.

### 2.10. Texture Assessment

Textural analysis was performed using a TA-XT Plus texture analyzer (Stable Micro Systems Ltd., Godalming, Surrey, UK). Warner-Bratzler and cylinder probes were used, and the operating parameters were 25 kg load cell, cutting speed 1.5 mm/s, piston retraction speed 10 mm/s, and distance 30 mm [30]. The meatballs were sectioned on the central axis, 24 h after preparation. Hardness (N), cohesiveness, and gumminess (N) were recorded.

### 2.11. Statistical Analysis

The data were statistically processed and expressed as mean ± standard deviation (SD) for each experimental batch and, for each storage time, performed in triplicate. The differences between batches were evaluated by one-way ANOVA, and, for the parameters analyzed according to storage time and addition level, two-way ANOVA was used. The comparison of means was performed by the post hoc Tukey test, significant differences being expressed by distinct letters, at a significance threshold of *p* < 0.05. For texture parameters, Pearson correlation coefficients (r) were also evaluated to identify linear associations and Spearman correlation coefficient (ρ) for confirmation.

## 3. Results and Discussion

### 3.1. Chemical Analysis of Samples

The chemical composition of the control sample (M) and the samples with the addition of wheat bran (G1, G2, and G3) is presented in Table 2. All chemical composition parameters were significantly influenced by batch membership, with the ANOVA indicating in all cases highly significant global differences (*p* < 0.001). The control batch stood out for the highest levels of proteins, lipids, and energy value, while G2 presented the highest water content, and G3 concentrated the highest values for the mineral substance. The Tukey test confirmed, for most parameters, a clear separation between batches, except for the ash content, where M, G1, and G2 formed a statistically homogeneous group.

The results highlight a progressive and clear reduction in protein, lipid, and energy content and an increase in minerals from the control to the G3 batch.

### 3.2. pH Assessment

**Figure 2:**
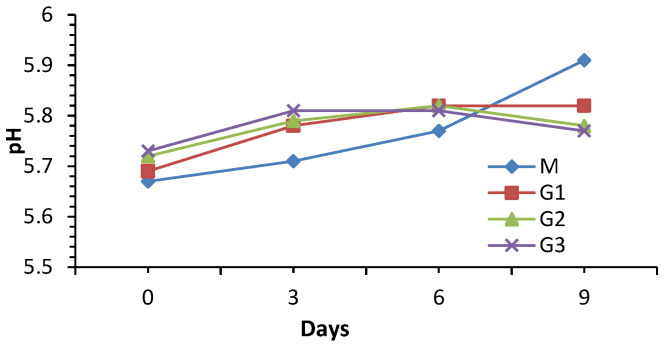
Variation in pH of the analyzed meatball samples supplemented with wheat bran (M—control sample; G1—sample with 5% wheat bran; G2—sample with 10% wheat bran; and G3—sample with 15% wheat bran).

The pH variation in beef patties with wheat bran additions (Figure 2) followed different evolutions for the control sample and for the samples with bran additions. While the control sample (M) presented a constantly increasing evolution of pH values, in the case of the G1, G2, and G3 samples, the pH increased up to a certain point, after which the values decreased or stagnated. The differences between the initial pH values (immediately after obtaining and cooling) were small (*p* > 0.05), the addition of wheat bran generating a slight increase in pH. Thus, the control sample presented a pH of 5.67, G1 had a pH of 5.69, G2 had a pH of 5.72, and G3 had a pH of 5.73.

For the addition of wheat bran to beef patties, increases in pH were reported from 6.01 ± 0.04 to 6.08 ± 0.02 [31], from 6.02 ± 0.02 to 6.26 ± 0.16 [10], and from 5.91 to 6.11 [32]. There are also studies reporting a decrease in pH with the increasing percentage of added wheat derivatives [33,34].

The pH of the control sample (M) increased significantly throughout the investigation period, reaching a final value of 5.91, while the pH of the G1 sample increased until the sixth day of storage, after which it remained constant until the ninth day, the final value being 5.82. The pH of sample G2 also increased until the sixth day, after which a slightly decreasing evolution followed. In the case of sample G3, the pH evolution had a slightly downward slope after the third day of storage, becoming more pronounced after the sixth day of storage. On day 9, the ANOVA revealed highly significant differences between batches (*p* < 0.001), confirmed by the Tukey test. At the end of the storage period, the control sample presented a significantly higher pH than all batches with added bran (*p* < 0.001), while G2 and G3 do not differ from each other in terms of registered pH (*p* > 0.005).

A similar evolution of decreasing pH during the last part of storage was reported not only for beef meatballs with the addition of wheat flour [31], and beef pasta with the addition of wheat germ powder [35], but also for turkey sausages in which apple fibers were introduced [34]. There are studies reporting a constant increase during storage of the pH of beef samples in which wheat bran was introduced [10].

The pH modification in meatball samples supplemented with plant derivatives was associated with protein denaturation and the formation of basic compounds that can generate an increase in pH during storage [13]. Other studies stated that the decreasing evolution during the last part of storage of samples with wheat bran could be explained by the presence of carbohydrates (especially dextrose) in wheat bran which represent a nutritional substrate for lactic acid bacteria [11,36].

### 3.3. Fiber Content Results

The values for total fiber content increased progressively from the control sample to G3, indicating a clear effect of the addition level on the total fiber content (Table 3). The values obtained in the present study, reported for dry matter, are close to the results of the other researchers. Yasarlar et al. [16] reported a concentration of 3.87%, 5.51%, and 7.48% fiber for cooked veal meatballs in which 5, 10, and 15% wheat bran, respectively, was incorporated. Other authors reported a lower fiber content that ranged from 1.09%, to 2.26%, to 3.02% (to 100 g sample) for samples that incorporated 6%, 8%, and 12% hydrated bran [37]. For chicken meatballs in which 10% wheat bran was added, the fiber value increased to 5.0 ± 0.54%, compared to the control sample in which the fiber was 0.43 ± 0.19% [13].

The lignin content follows the same increasing pattern (M < G1 < G2 < G3), which confirms the increase in the analyzed fraction with the increase in the proportion of added wheat bran, the statistical analysis showing significant differences for all pairwise comparisons (*p* < 0.001). In wheat bran, the lignin content represents 5.4–6.1/100 g dry matter bran [38]. In the samples analyzed in the present study, the lignin concentration increased with the increase in the added bran amount, from 0.125 ± 0.011% for control, to 4.420 ± 0.368% for G3.

For cellulose, a consistent increase can also be observed from M to G3. The effect of bran addition is significant (*p* < 0.001), and the Tukey test confirms significant differences between all groups. Cellulose, which is reported in the literature as 19.5–20.3% per 100 g dry matter of wheat bran [38], varied in our study from 0.407 ± 0.005% for the control sample to 12.625 ± 0.362% for sample G3.

For all three parameters (total fiber, lignin, and cellulose), statistical analysis revealed significant differences (*p* < 0.001) and confirmed a clear separation, with a monotonic increase in values from M to G3.

### 3.4. Mineral Content of Beef Patties

The mineral content of the analyzed mixtures, as can be seen from Table 4, changed in correlation with the mineral content of the raw materials and ingredients used.

Regarding to beef, de Freitas et al. [39] reported a mineral content for the Longissimus dorsi muscle of 1.08 ± 0.01 g/100 g. This concentration was found to be different on the ecosystem characteristics, with sodium (5216 mg/kg) and calcium (1020.2 mg/kg) values being higher in the extensive system [40].

Wheat bran and the spices used influenced, in turn, the mineral profile of the investigated samples. The average mineral content of wheat bran, based on dry weight, was reported at 2.80 ± 0.15% [41]. However, there are studies that show a mineral content of 6.2% [42]. Bran is also an important source of minerals such as potassium (1340 mg/100 g), phosphorus (1142 mg/100 g), magnesium (480 mg/100 g), calcium (67 mg/100 g), iron (16 mg/100 g), and manganese (13 mg/100 g) [42].

In the present study, we found significant variations in mineral concentrations: decreases were recorded for Na (13.76%) and Zn (18.76%); insignificant increases for K (0.21%) and Fe (3.335%); and significant increases for Mg (117.55%), P (15.39%), Ca (32.25%), Cu (90.3%), and Cr (111.81%). Mn increased from 1.63 mg/Kg to 34.058 mg/kg. Mineral analysis highlights a clear effect of the bran addition on most of the investigated elements. For Mg, Ca, Mn, Cu, and Cr, the ANOVA indicated significant differences between samples (*p* < 0.001), and the Tukey test confirms clear separations. In contrast, for K and Fe no significant differences were observed (*p* > 0.05), which suggests a relative stability of these elements with respect to the addition of bran. Na, P, and Zn show significant but uneven changes.

The increases in P, Mg, Ca, and Fe concentrations following the addition of fiber are in agreement with the data presented by other authors [42]. Moreover, the decrease in Na and Zn concentrations with the increase in the percentage of bran in the samples analyzed by us correlates with a low content of these elements in bran.

### 3.5. Fatty Acid Levels in Beef Patties

The lipid profile of meatballs depends on the lipid content of the beef, which varies depending on the fattening stage between 3.0 and 20.0%, these being mainly composed of neutral glycerides, small amounts of phospholipids 0.5–0.9% and approximately 0.8% cholesterol [30]. The PUFA content of beef ranges from 3 to 10% of total fatty acids, whereas wheat bran contains 3.5–5% fat, of which 60–70% are PUFAs, expressed as a proportion of total fatty acids [43]. The addition of wheat bran to the composition of beef patties determined significant changes in the lipid profile of the samples, with a general tendency to reduce the saturated fraction and increase the polyunsaturated fractions from the control sample to the experimental variants with wheat bran (Table 5).

Regarding the total saturated fatty acid (SFA) concentration, a proportional decrease can be observed with the increase in the addition of bran, results also recorded by other authors (Yilmaz; Talukder) [13,32]. The SFA concentration decreased from 45.78 g/100 g FA for control, to 43.56 g/100 g FA for G3, the differences being significant only between M and batches G2 (*p* < 0.05) and G3 (*p* < 0.01), while G1 did not present significant differences compared to the other samples. Similar concentrations of SFA have been recorded in beef samples by other researchers [44]. Beefburger in which the beef fat was partially replaced with wheat bran fibers and olive oil recorded a decrease in SFAs, from 51.6 g/100 g fat to 20.54 g/100 g fat [44]. Obtaining dietetic chicken meatballs by incorporating wheat bran generated a decrease in SFAs from 35.1% to 34.3%, an increase in UFSAs from 64.9% to 65.7%, and an increase in the UFSA/FSA ratio from 1.8 to 1.9 [13].

Unlike other authors who recorded increases in MUFAs, from 43.66 g/100 g fat—control to 44.8 g/100 g fat—sample with 20% wheat bran [32], and from 42.29 g/100 g fat to 66.61 g/100 g fat, under the conditions of using olive oil [44], in the case of our study, the total MUFA concentration decreased by 9.63% upon the addition of 15% wheat bran. The ∑MUFA remained practically constant between M, G1, and G2, but decreased significantly in G3 (*p* < 0.001), which suggests that the major modification of this fraction occurs predominantly at higher contents of bran (the G3 sample with the 15% addition).

The increase in the added wheat bran concentration determined a very clear statistical increase (*p* < 0.001) of the PUFA fraction by 14.7% for G1, 31.12% for G2, and 54.08% for G3, respectively, similar increases being recorded by other authors [13,32,44]. The same dynamics were observed for ∑n3 and ∑n6.

Among the saturated fatty acids in the control sample, the highest values were found for C16:0, C18:0, and C14:0, results also recorded by other authors for beef [43,44]. The same acids also presented the highest content for the samples with the wheat bran addition. With the exception of C15:0 and C17:0 acids, which recorded increases, all other saturated fatty acids recorded decreases in the wheat bran batches compared to the control. Within the saturated acids, C10:0 and C12:0 decreased continuously and significantly in the case of all samples, C14:0 was significantly higher in the control, and C16:0 had a downward trend without significant differences (*p* > 0.05). For C17:0, the G3 sample registered the maximum value and was clearly separated from all other groups (*p* < 0.001).

C18:1n-9 had the highest proportion of monounsaturated fatty acids (Table 5), for all samples analyzed, its concentration decreasing from 39.09 g/100 g FA for the control sample, to 35.20 g/100 g FA for the G3 sample (*p* < 0.001). In contrast, C18:1n-7 increased gradually and significantly between all groups. For beef, C18:1 has also been reported in other studies as being the majority [43,45]. In the context of other experimental formulations, such as beefburger with olive oil and wheat bran fiber, C18:1n-9 recorded substantial increases from 38.97 g/100 g fat to 65.17 g/100 g fat [44].

For monounsaturated fatty acids, C14:1 and C17:1 showed significant reductions from the control to samples with wheat bran in their composition (*p* < 0.001), except for the G2-G3 comparison for C14:1, where the values remained statistically similar (*p* > 0.05). C16:1 was significantly higher in the control than in all treated groups (*p* < 0.001).

Among the polyunsaturated fatty acids, C18:2 cis had the highest concentration in all the analyzed samples, its concentration increasing with an increasing concentration of added wheat bran, by 8.49% for G1, 15.37% for G2, and 38.19% for G3, respectively. The increase in the polyunsaturated fatty acid content with an increasing fiber addition was also reported by other authors [32,44]. C18:2 cis (linoleic) and C18:3 cis n3 (α-linolenic) increased significantly, without statistical overlap. Moreover, C18:3 n6 followed the same ascending pattern, with significant differences for all samples (*p* < 0.001). These results support the idea of an improvement in nutritional quality by increasing the addition of bran.

The addition of wheat bran to the structure of beef patties determined the increase in the PUFA/SFA and UFA/SFA ratios. Thus, PUFA/SFA increased from 0.20 for the control to 0.27 for G3, while UFA/SFA increased significantly from 1.17 for M to 1.25 for G3. Similar tendencies were found in other studies. For PUFA/SFA, values of 0.21 for the control and 0.34 for the wheat bran sample [32], 0.28 for the control and 0.38 for the bran sample [10], and 0.07 for the control and 0.31 for the oil and bran sample [44] were reported. For the UFA/SFA ratio, for beef formulations, other authors recorded values of 0.89–1.54 [44], and 0.99–1.06 [32].

This increase may be associated with an improvement in the nutritional quality of meatballs, with PUFA and MUFA sequences generally considered beneficial to the body [39].

### 3.6. Amino Acid Levels in Beef Patties

The amino acid profile highlights a consistent effect of the percentage of wheat bran added to the meatballs on most parameters, as can be seen in Table 6. For almost all quantified amino acids, the control sample presented the highest average values, followed by the G1 and G2 samples, while G3 generally presented the lowest values. For most amino acids (aspartic acid, glutamic acid, alanine, arginine, phenylalanine, glycine, isoleucine, histidine, leucine, methionine, serine, tyrosine, and valine), the Tukey analysis showed that the control sample differed significantly from all samples with added wheat bran (G1, G2, and G3), while the differences between G1, G2, and G3 were not significant (*p* > 0.0.5). For the control sample (M), glutamic acid (5.14 ± 0.54 g/100 g), aspartic acid (3.20 ± 0.36 g/100 g), and lysine (3.19 ± 0.36 g/100 g) dominated, their high share dictating the same dominance in the samples with added wheat bran. The products obtained from cereal-based meat analogues are deficient in lysine, which is the limiting amino acid for whole grains (Wang) [45] However, the mixed use of meat and cereal derivatives helps to balance the amino acid ratio and obtain products with a high nutritional value.

Evaluating the content in cystine + cysteine and threonine, it can be observed that G1 presented a transition profile, indicating that it does not differ significantly from the control, G2, and G3, while G2 and G3 showed significant differences (*p* < 0.05) and distinctly significant differences (*p* < 0.01) from the control, respectively.

For proline, only the difference between M and G3 was significant (*p* < 0.05), suggesting an obvious response only at the maximum level of addition. Lysine and total free amino acids revealed the most suggestive separation patterns. In the case of lysine, G3 was significantly lower than the control (*p* < 0.001) and G1 (*p* < 0.05), and G2 occupied an intermediate position, indicating a gradual increase in differences. For total free amino acids, the control sample was clearly superior to all groups, the differences being very significant (*p* < 0.001); G1 and G2 did not differ between them (*p* > 0.05), while G3 was significantly lower than both (*p* < 0.001), confirming an overall reduction in free amino acids in the group with the highest percentage of bran addition. Hydroxyproline was reported below the limit of quantification (*p* < 0.01) in all groups; consequently, the ANOVA/Tukey analysis was not performed for this parameter. The results indicate that the percentage of bran addition determined a differential decrease in the proportion of amino acids in the analyzed groups. Results consistent with those obtained in our study were also reported by other authors [2,46].

For various chicken, beef, and pork burger formulations, total amino acid concentrations ranging from 264.82 mg/g protein to 182.64 mg/g protein were reported, while, for various plant-based burger patties, values between 43.42 mg/g and 248.1 mg/g were recorded [2]. For Aubrac cattle (*Longissimus dorsi*), total amino acids of 30.59 ± 0.49 g/100 g protein for males and 25.97 ± 0.64 g/100 g protein for females were reported [46].

Glutamic and aspartic acids, identified in the present study in a high proportion in all investigated samples, contribute to defining the aroma of products, participating in Maillard reactions and hydrophobic interactions during heating [47] and conferring the umami taste to meat products [45]. The free amino acids alanine, glycine, threonine, and serine contribute to the sweet taste, while valine, methionine, leucine, phenylalanine, histidine, tryptophan, arginine, and isoleucine contribute to the bitter taste [48].

### 3.7. Evolution of Antioxidant Activity of Meatballs with Added Wheat Bran

The destruction of the meat structure during processing, the application of heat treatments, the addition of salt, and the presence of air, water, and large amounts of unsaturated lipids are factors that support the oxidation of lipids in meat emulsions [49]. However, meat and meat products contain substances such as glutathionecarnosine, and anserine, whose antioxidant properties can be highlighted by the DPPH test, a common chemical test that measures the ability of a substance to capture free radicals [50]. This capacity varies depending on the species due to the sequence of amino acids in proteins, as well as the presence of proteinases that cleave proteins with the formation of peptides that can donate electrons and interact with free radicals to reduce and/or block chain reactions [1].

On the other hand, whole grains contain phenolic acids and flavonoids with strong antioxidant properties and which have beneficial effects on health [8,11]. These phenolic compounds can reduce oxidative stress in the body, have good free radical scavenging activity [45], and have the ability to reduce blood sugar [9].

Analyzing the results obtained for antioxidant activity (Table 7), it can be observed that, for all days of storage, significant differences were recorded between samples (*p* < 0.001), which confirms the influence of the wheat bran addition on the antioxidant activity. At the time of sample production, the antioxidant activity recorded significant increases for all samples in which wheat bran was introduced compared to the control (M), the differences being significant between all compared pairs (*p* < 0.001).

During storage, the evolution of the antioxidant capacity was different. The evolution of the DPPH scavenging ability (Table 7) increased for the control sample up to 3 days of storage, after which it decreased for the rest of the storage period. In the case of samples with bran additions (G1, G2, and G3), DPPH-RSA registered a rather linear decreasing tendency in time. However, it can be seen that increasing bran percentages resulted in a significant increase in the scavenging ability of the sample extracts. Compared to sample M, samples G1, G2, and G3 recorded lower antioxidant activities throughout the storage period, as can be seen in Table 7. Compared to day 0, the response no longer strictly followed the increasing model with the addition level, suggesting the temporal dynamics of antioxidant compounds and an interaction between treatment and storage duration.

At the end of storage (day 9), a general decrease in antioxidant activity was observed compared to previous days, suggesting the progressive degradation of antioxidant compounds over time. The control (M) remained superior to the other samples, with significant differences (*p* < 0.001). G3 occupied an intermediate position, with significant differences compared to G1 (*p* < 0.01) and G2 (*p* < 0.001), and G1 and G2 did not differ significantly (*p* > 0.05).

The increase in the DPPH of sample M in the first days of storage can be explained by the proteolytic enzymes (cathepsins and collagenase) remaining after the heat treatment that will act on beef proteins with the formation of small peptides with antioxidant activity, as reported by other authors [49,51]. However, with an increasing storage time, oxidative reactions become predominant, which leads to the consumption of antioxidant compounds and the decrease in DPPH [50]. The high temperature of the heat treatment generates Maillard reactions, the compounds formed having an important antioxidant effect, by converting DPPH into a stable form of DPPH-H, capturing hydroxyl radicals and inhibiting lipid peroxidation [52]. Another explanation could be that the antioxidants present in meat, which are formed following heat treatment, have a high thermal resistance and are more stable over time compared to those in cereals [50].

The continuous decrease in antioxidant activity for samples G1, G2, and G3, compared to M, can be explained by the oxidation and progressive depletion of phenolic compounds naturally present in the bran and by the complexity of the emulsion formed, and by the reactions that are established between the components of the system—tannins can form complexes with meat proteins, iron and copper in meat can reduce the activity of polyphenols, and, in an acidic environment, polyphenols form less active complexes [52].

The decrease in antioxidant activity with increasing storage time was also mentioned by other researchers [49,50].

### 3.8. Variation of Peroxide Value

**Figure 3:**
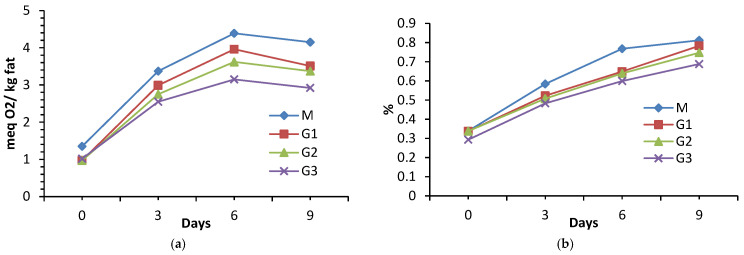
Evolution of fat degradation indicators during storage of meatballs containing wheat bran: (**a**) variation in the peroxide value; and (**b**) variation in free acidity.

Analyzing the evolution of the peroxide index (PV) during storage, it can be seen that it increased for all experimental variants, reaching maximum values on the sixth day of storage, after which the evolution was decreasing (Figure 3a). The increase in PV occurs as a result of the oxidation of unsaturated fats, and the decrease is due to the fact that hydroperoxides are unstable compounds, which, in advanced stages of oxidation, decompose into secondary oxidation products (aldehydes, alkenes, ketones, and alcohols that affect the smell of meat), the decomposition rate being higher than the formation rate [8,10,43].

At the time of sample production, PV presented significantly different values (*p* < 0.05) for all samples (1.35 ± 0.03 meq O_2_/kg fat for M; 0.97 ± 0.22 meq O_2_/kg fat for G1; 0.97 ± 0.02 meq O_2_/kg fat for G2; and 1.02 ± 0.10 meq O_2_/kg fat for G3); however, the effect of adding wheat bran on PV became even more visible on days 3, 6, and 9 (*p* < 0.001). The control values were superior to the G1, G2, and G3 variants at all experimental times, the differences becoming more pronounced with increasing storage time. This indicates a higher susceptibility of the control sample to lipid oxidation in the absence of vegetable compounds.

From day 3, the differences between M and G1, G2, and G3 become more pronounced, the control presenting values significantly higher than G1, G2 (*p* < 0.01), and G3 (*p* < 0.001), becoming, on day 9, distinctly significant compared to G1 (*p* < 0.01) and increasing even more compared to G2 and G3 (*p* < 0.001). On day 9, G3 presented values distinctly significantly lower than G1 (*p* < 0.01) and significantly lower than G2 (*p* < 0.001).

The highest amplitude in the increase in the peroxide index was observed in the control sample, with a maximum PV of 4.39 ± 0.15 meq O_2_/kg fat on the sixth day of storage, the lowest value being recorded for sample G3, which had a PV of 3.15 ± 0.19 meq O_2_/kg fat, measured at the same time. At the end of the investigated period, the lowest PV value was also for sample G3 (2.92 ± 0.06 meq O_2_/kg fat), followed by samples G2 (3.37 ± 0.06 meq O_2_/kg fat) and G1 (3.51 ± 0.13 meq O_2_/kg fat), the control having the highest PV value (4.15 ± 0.13 meq O_2_/kg fat).

Statistical analysis confirmed that the addition of wheat bran significantly influenced the evolution of the peroxide index of beef patties during storage, the effect being dependent on both the concentration of the ingredient and the duration of storage. Variants with higher levels of bran showed a superior oxidative stability, indicating the potential of using this ingredient to improve the quality and shelf life of meat products. The factorial ANOVA analysis also revealed a significant interaction between storage time and the proportion of wheat bran (*p* = 0.004), indicating that the effect of wheat flour on lipid oxidation is not constant over time, but increases as the storage duration increases.

The variation in the peroxide index of the samples that incorporated wheat bran correlates with the evolution of the antioxidant activity. The samples that registered a low antioxidant activity had high PV values.

Cantele et al. [53], whose results are in agreement with ours, showed that, at the time of production, the beef samples treated with alkylresorcinols extracted from wheat bran had a hydroperoxide level of 0.67 ± 0.10 meqO_2_/kg fat, much lower compared to the control, which had 1.04 ± 0.27 meqO_2_/kg fat. This oxidation stability is justified by the efficient action of alkylresorcinols in eliminating free radicals and chelating metals (ferrous ions). The incorporation of wheat bran of different grain sizes into beef patties resulted in a decrease in PV, with this decrease being greater the finer the bran was ground [8]. Khalid A. et al. [10] demonstrated a decrease in PV in low-fat beef products containing 3% wheat bran (the values recorded were 0.69 meq/kg for the control sample, 0.54 meq/kg for the bran sample, and 0.39 meq/kg for the 3% bran and 4.5% wheat germ oil sample), with the cooked samples having a higher PV than the raw ones. Jamaly et al. [31] stated that beef patties containing 5, 10, and 15% wheat flour had lower PV values than the control.

One aspect to discuss is the situation of the control, which, although, from the third day of storage, has a higher antioxidant activity than the bran samples, registered the highest PV. The increase in oxidation stability may be favored by the presence of phenolic acids and flavonoids in wheat bran or by the presence of antioxidant substances in meat: vitamins (Vitamin E and α-tocopherol), peptides (carnosine, anserine, and glutathione), and enzymes (superoxide dismutase, catalase, and glutathione peroxidase) [54]. On the other hand, the presence of a high nonheme iron content in cooked beef may act as a prooxidant and is responsible for the high susceptibility of lipid oxidation [55]. It is considered that the most important pro-oxidant factors in lipid oxidation are ferric heme pigments, and, during storage under refrigerated conditions, heme degradation and an increase in non-heme iron content were observed. There is a synergy between heme and lipid oxidation, with ferric heme favoring lipid oxidation, and the resulting oxidation compounds affecting heme and favoring the detachment of iron ions [56].

Another aspect that must be taken into account is the initial level of fat oxidation, since peroxides already formed cannot be annihilated by antioxidants introduced into the system, which protect the meat from the action of free radicals, or metals that catalyze the oxidation process [43].

Although the degree of unsaturation of fats is very important in the development of oxidation, there are researchers who highlight that the content of phospholipids is essential in determining oxidation and rancidity [57]. Technological processes, such as chopping, cooking, and storage, also influence the degree of fat oxidation [57]. The species and way of raising animals can influence the PV value, with free-range raising favoring antioxidant activity in the meat [58]. Funaro et al. [59] showed that free-range and conventional rearing systems generated a similar PV for raw meat (<1.3 meq O_2_/kg), but cooked free-range chicken meat had a lower PV.

### 3.9. Variation of Free Acidity

The results regarding free fatty acid evolution with storage time are presented in Figure 3b. It can be observed that FFAs (expresses as % of oleic acid) decreased with the increase in the percentage of wheat bran added and, for each experimental group, increased during the storage period. The FFA values at the time of sample obtaining were 0.3386 (M), 0.3372 (G1), 0.3384 (G2), and 0.2926 (G3), respectively, with significant differences between M and samples with bran in their composition (*p* < 0.001). At the end of the storage period, the free acidity reached values of 0.8117 (M), 0.7833 (G1), 0.747 (G2), and 0.6884 (G3). Repeatedly, G1 and G2 did not differ significantly from each other (days 0, 3, and 6, *p* = 0.05), but both differed from G3, indicating that the 15% addition level produced a more pronounced reduction in acidity. At day 9, significant differences appeared between all groups, indicating that, in the long term, the separation between the addition levels became more evident (*p* < 0.001). The lower FFA values for the samples with added wheat bran showed their ability to limit lipolytic activity in the meat matrix. This evolution can be explained by the ability of fibers to retain water and limit enzyme mobility, as well as by the antioxidant activity, which can inhibit endogenous lipases and protect glycerides from oxidation [47].

Similar observations were also made by other researchers. At the time of sample production, the FFA values for beef patties were reported to be 0.3, with the addition of 15% wheat flour decreasing the FFAs to 0.27 [31], similar to our study. Kim et al. [60] reported an increase in the acidity index of raw beef from 0.15 ± 0.05 mg KOH/g to 0.54 ± 0.05 mg KOH/g after cooking. Storing aged beef for 13 days increased the acidity index from 0.8 to 5.0 [61]. The addition of 3% Salicornia perennans powder to beef sausage paste decreased the acidity index from 5.5 ± 0.3 mg KOH/g for the control sample to 4.6 ± 0.3 mg KOH/g after 10 days of refrigerated storage [21]. Beef and turkey meatballs (80:20) with a multicomponent creal supplement (rice, buckwheat, oats, and corn) recorded, after five days of storage, values of the acidity index of 1.90–2.14 mg KOH/g, while the control sample recorded values of 2.33 mg KOH/g [47].

There is a correlation between the indicators of fat degradation, FFAs, and POV having similar evolutions and higher values, for both parameters, recorded for M and with increasing storage time. Both the acidity index and the peroxide index are parameters that represent a quantification of the initial phases of lipid oxidation [60]. Under the action of lipases and in the presence of water, triglycerides undergo hydrolysis processes, after which they are decomposed into glycerol, mono, diglycerides, and fatty acids (FFAs). Studies showed that the fatty acids resulting from the lipolysis process could be the basis for the oxidative degradation of lipids and the formation of volatile compounds responsible for the aroma of meat. Aldehydes, acids, and lactones, resulting from fatty acid oxidation and ketones formed mainly from amino acid degradation, lipid oxidation, and Maillard reactions, are important in determining the aroma in oxidized beef fat [62]. A vacuum applied in the heat treatments of beef can prevent secondary lipid oxidation, although there is no effect on the early stages of lipid oxidation [60].

### 3.10. Analysis of the Textural Profile of the Samples

The evolution of the textural parameters for beef meatballs in which wheat bran was incorporated is presented in Table 8. The increase in the percentage of wheat bran and the reduction in the proportion of beef in the meat pastes intended for obtaining meatballs, respectively, generated very significant reductions (*p* < 0.001) for all textural parameters investigated. Thus, at the time of sample production, in comparison with the control (M), which recorded a hardness of 42.903 N, the hardness of samples G1, G2, and G3 decreased to 31.303 N, 27.372 N, and 21.303 N, respectively. The reduction in hardness can be explained by the interposition of fibers between the meat proteins, with which they establish bonds, thus weakening the protein matrix of the product [34]. On the other hand, fibers have the ability to stabilize the meat emulsion and retain water and fat, with a direct effect on the texture [15].

The introduction of apple pomace powder into the structure of turkey sausages generated a decrease in hardness from 76.73 ± 10.64 N to 59.7 ± 6.04 N [34]. The use of red algae in the injection brine for pork loin determined a decrease in hardness from 33.14 ± 2.95 N to 19.69 ± 2.82 N [63]. The incorporation of *Cetraria islandica* in pork products generated a decrease in hardness from 21.56 ± 2.20 N to 8.87 ± 0.54 N [64].

There are also studies that show different developments from those in our work. Replacing fat with wheat bran in beef sausages generated increased hardness, cohesiveness, and gumminess [5,19], the fibers being able to establish denser structures than fat. Obtaining beef burgers by replacing fat with wheat bran led to an increased hardness from 42 ± 5 N to 45 ± 7 N [33].

For cohesiveness, significant differences were recorded between the analyzed samples, with values ranging between 0.511 (M) and 0.249 (G3). Decreases in cohesiveness upon the incorporation of different fibers in meat emulsions were also recorded by other researchers [33,34], but, at the same time, there are also studies reporting increases in cohesiveness [5,19].

Significant differences were also recorded in the case of the gumminess variation, the decrease being proportional to the proportion of wheat bran added. The values recorded for gumminess were 22.337 N (M), 19.641N (G1), 16.433 N (G2), and 13.134 N (G3), respectively. The decrease in gumminess is consistent and concomitant with the reduction in hardness and cohesiveness and suggests a texture that is less resistant to chewing as the addition of bran increases. Similar developments were reported also for turkey sausages with apple fibers [34].

## 4. Conclusions

The study highlights the significant impact of the addition of wheat bran on the nutritional and functional profile of beef meatballs. The fortification of beef meatballs led to a significant increase in fiber content, contributing to the improvement in the dietary value of the product. At the same time, an increase in the proportion of polyunsaturated fatty acids (PUFAs) was observed, a favorable aspect from a nutritional perspective. However, the addition of bran caused a decrease in the amino acid content, suggesting a protein dilution effect. The addition of bran also led to an improvement in texture.

Statistical analysis confirmed and increasing oxidative stability and hydrolytic stability with increasing wheat bran concentration, indicating the potential of this ingredient in improving the shelf life of meat products.

Therefore, in the context of industrial implementation, it is essential to further investigate the shelf life under various packaging and storage conditions, as well as to assess the sensory attributes and consumer acceptability of the product.

## Figures and Tables

**Table 1:** Forming emulsions for obtaining beef meatballs with wheat bran.

Ingredients, g	Samples			
	M	G1	G2	G3
Beef	100	95	90	85
Wheat bran	0	5	10	15
Hydrating water for meat	10	9.5	9.0	8.5
Hydrating water for wheat bran	0	25	50	75
Spices	1	1	1	1
NaCl	1	1	1	1

M—control sample (no additions); G1—beef emulsion with 5% wheat bran; G2—beef emulsion with 10% wheat bran; G3—beef emulsion with 15% wheat bran.

**Table 2:** Dynamics and statistical significance of values for chemical composition of beef meatballs supplemented with wheat bran.

Parameter	Sample				SEM	*p*-Value
	M	G1	G2	G3		
Moisture, %	68.193 ± 0.316 ^c^	70.830 ± 0.296 ^b^	72.760 ± 0.316 ^a^	68.507 ± 0.217 ^c^	0.167	<0.001
Protein, %	22.160 ± 0.233 ^a^	17.880 ± 0.115 ^b^	15.970 ± 0.255 ^d^	16.440 ± 0.202 ^c^	0.120	<0.001
Fat, %	6.800 ± 0.157 ^a^	5.710 ± 0.111 ^b^	4.980 ± 0.108 ^c^	4.220 ± 0.108 ^d^	0.071	<0.001
Ash, %	2.060 ± 0.027 ^b^	2.040 ± 0.019 ^b^	2.020 ± 0.016 ^b^	2.590 ± 0.030 ^a^	0.014	<0.001
Energy, kcal/100 g	154.100 ± 1.881 ^a^	126.600 ± 1.534 ^b^	111.790 ± 1.486 ^c^	106.650 ± 1.777 ^d^	0.968	<0.001

Values are expressed as mean ± standard deviation; SEM—common standard error of the means; *p*-value—global test (ANOVA) for the comparison between the 4 batches for the same parameter; M—control sample; G1—sample with 5% wheat bran; G2—sample with 10% wheat bran; and G3—sample with 15% wheat bran. Values within the same row bearing distinct letters denote statistically significant variations (*p* < 0.05).

**Table 3:** Fiber content of meatball samples.

Parameter	Samples				SEM	*p*-Value
	M	G1	G2	G3		
Total fiber, g/100 g DM	0.532 ± 0.016 ^d^	7.493 ± 0.301 ^c^	10.807 ± 0.674 ^b^	17.045 ± 0.730 ^a^	0.030	<0.001
Lignin, g/100 g DM	0.125 ± 0.011 ^d^	2.313 ± 0.182 ^c^	3.073 ± 0.255 ^b^	4.420 ± 0.368 ^a^	0.014	<0.001
Cellulose, g/100 g DM	0.407 ± 0.005 ^d^	5.180 ± 0.119 ^c^	7.734 ± 0.419 ^b^	12.625 ± 0.362 ^a^	0.016	<0.001

SEM—common standard error of the means; *p*-value—global test (ANOVA) for the comparison between the 4 batches for the same parameter; M—control sample; G1—sample with 5% WB; G2—sample with 10% WB; G3—sample with 15% WB; and DM—dry matter. Values within the same row bearing distinct letters denote statistically significant variations (*p* < 0.05).

**Table 4:** Macro- and microelement content of meatball samples supplemented with wheat bran.

Parameter	Samples				SEM	*p*-Value ANOVA
	M	G1	G2	G3		
Na, mg/100 g	1244.406 ± 13.600 ^a^	1210.047 ± 33.760 ^b^	1137.226 ± 82.292 ^bc^	1073.218 ± 33.036 ^c^	27.670	0.0098
Mg, mg/100 g	73.219 ± 1.525 ^d^	109.965 ± 3.703 ^c^	140.850 ± 8.529 ^b^	159.287 ± 1699 ^a^	2.763	<0.001
P, mg/100 g	632.242 ± 4.038 ^c^	659.084 ± 19.446 ^bc^	691.579 ± 53.977 ^ab^	729.568 ± 23.930 ^a^	17.983	0.025
K, mg/100 g	1214.859 ± 20.398 ^a^	1236.541 ± 40.994 ^a^	1195.31 ± 87.290 ^a^	1217.382 ± 4.406 ^a^	28.483	0.79
Ca, mg/100 g	48.914 ± 1.678 ^c^	71.694 ± 2.853 ^a^	70.535 ± 4.573 ^a^	64.688 ± 0.197 ^b^	1.631	<0.001
Fe, mg/100 g	7.785 ± 0.121 ^a^	7.644 ± 0.344 ^a^	8.032 ± 0.474 ^a^	8.046 ± 0.141 ^a^	0.177	0.363
Mn, mg/Kg	1.630 ± 0.037 ^d^	15.835 ± 0.619 ^c^	27.129 ± 1.696 ^b^	34.058 ± 0.628 ^a^	0.552	<0.001
Cu, mg/Kg	2.618 ± 0.092 ^d^	3.653 ± 0.073 ^c^	4.504 ± 0.284 ^b^	4.982 ± 0.039 ^a^	0.090	<0.001
Cr, mg/Kg	0.364 ± 0.015 ^d^	0.702 ± 0.040 ^b^	0.620 ± 0.014 ^c^	0.771 ± 0.012 ^a^	0.013	<0.001
Zn, mg/Kg	204.899 ± 20.302 ^a^	193.961 ± 12.822 ^a^	186.250 ± 2.968 ^ab^	166.456 ± 9.557 ^b^	7.509	0.0363

SEM—common standard error of the means; *p*-value—global test (ANOVA) for the comparison between the 4 batches for the same parameter; M—control sample; G1—sample with 5% WB; G2—sample with 10% WB; and G3—sample with 15% WB. Values within the same row bearing distinct letters denote statistically significant variations (*p* < 0.05).

**Table 5:** Wheat bran effect on the lipid profile of beef patties (g/100 g FA).

Parameter	Samples				SEM	*p*-Value ANOVA
	M	G1	G2	G3		
∑SFA	45.783 ± 0.820 ^a^	44.630 ± 0.804 ^ab^	44.049 ± 0.795 ^b^	43.567 ± 0.788 ^b^	0.463	0.0450
∑MUFA	44.351 ± 0.801 ^a^	44.363 ± 0.801 ^a^	43.580 ± 0.785 ^a^	40.083 ± 0.726 ^b^	0.450	0.0004
∑PUFA	9.322 ± 0.165 ^d^	10.690 ± 0.193 ^c^	12.221 ± 0.222 ^b^	14.361 ± 0.258 ^a^	0.122	0.0001
∑n3	0.411 ± 0.006 ^d^	0.451 ± 0.006 ^c^	0.911 ± 0.015 ^b^	0.961 ± 0.016 ^a^	0.007	0.0001
∑n6	8.720 ± 0.157 ^d^	9.460 ± 0.171 ^c^	10.060 ± 0.181 ^b^	12.050 ± 0.217 ^a^	0.106	0.0001
C10:0	0.460 ± 0.008 ^a^	0.300 ± 0.005 ^b^	0.140 ± 0.002 ^c^	0.050 ± 0.000 ^d^	0.003	0.0001
C12:0	0.720 ± 0.013 ^a^	0.510 ± 0.010 ^b^	0.490 ± 0.009 ^c^	0.380 ± 0.007 ^d^	0.006	0.0001
C14:0	3.170 ± 0.057 ^a^	2.810 ± 0.050 ^b^	2.570 ± 0.046 ^c^	2.660 ± 0.048 ^c^	0.029	0.0001
C14:1	0.590 ± 0.010 ^a^	0.471 ± 0.007 ^b^	0.341 ± 0.005 ^c^	0.350 ± 0.006 ^c^	0.004	0.0001
C15:0	0.450 ± 0.008 ^d^	0.510 ± 0.009 ^c^	0.540 ± 0.010 ^b^	0.830 ± 0.015 ^a^	0.006	0.0001
C16:0	24.972 ± 0.447 ^a^	24.691 ± 0.444 ^a^	24.311 ± 0.437 ^a^	24.172 ± 0.433 ^a^	0.254	0.1859
C16:1	3.681 ± 0.065 ^a^	2.710 ± 0.048 ^b^	2.640 ± 0.047 ^bc^	2.570 ± 0.046 ^c^	0.030	0.0001
C17:0	2.140 ± 0.039 ^bc^	2.180 ± 0.040 ^b^	2.080 ± 0.038 ^c^	2.580 ± 0.047 ^a^	0.024	0.0001
C17:1	0.760 ± 0.014 ^a^	0.580 ± 0.010 ^b^	0.510 ± 0.009 ^c^	0.440 ± 0.007 ^d^	0.006	0.0001
C18:0	13.451 ± 0.241 ^a^	13.720 ± 0.247 ^a^	13.381 ± 0.239 ^ab^	12.930 ± 0.233 ^b^	0.139	0.0229
C18:1n-9	39.091 ± 0.704 ^a^	39.360 ± 0.709 ^a^	38.520 ± 0.694 ^a^	35.201 ± 0.633 ^b^	0.396	0.0002
C18:1n-7	1.070 ± 0.019 ^d^	1.381 ± 0.025 ^c^	1.450 ± 0.026 ^b^	1.560 ± 0.028 ^a^	0.014	0.0001
C18:2 cis	8.720 ± 0.157 ^d^	9.460 ± 0.170 ^c^	10.060 ± 0.180 ^b^	12.050 ± 0.218 ^a^	0.106	0.0001
C18:3 cis n3	0.410 ± 0.007 ^d^	0.450 ± 0.008 ^c^	0.910 ± 0.016 ^b^	0.960 ± 0.017 ^a^	0.008	0.0001
C18:3 n6	0.250 ± 0.004 ^d^	0.380 ± 0.007 ^c^	0.450 ± 0.008 ^b^	0.510 ± 0.009 ^a^	0.004	0.0001
PUFA/SFA	0.200 ± 0.004 ^d^	0.210 ± 0.004 ^c^	0.230 ± 0.004 ^b^	0.270 ± 0.005 ^a^	0.002	0.0001
UFA/SFA	1.170 ± 0.021 ^b^	1.230 ± 0.022 ^a^	1.270 ± 0.023 ^a^	1.250 ± 0.022 ^a^	0.013	0.0027

SEM—common standard error of the means; *p*-value—global test (ANOVA) for the comparison between the 4 batches for the same parameter; M—control sample; G1—sample with 5% WB; G2—sample with 10% WB; and G3—sample with 15% WB. Values within the same row bearing distinct letters denote statistically significant variations (*p* < 0.05).

**Table 6:** Wheat bran addition effect on the protein profile of beef patties (g/100 g protein).

Parameter	Samples				SEM	*p*-Value ANOVA
	M	G1	G2	G3		
Aspartic acid	3.20 ± 0.36 ^a^	2.36 ± 0.29 ^b^	2.23 ± 0.28 ^b^	1.82 ± 0.25 ^c^	0.172	0.0030
Glutamic acid	5.14 ± 0.54 ^a^	3.91 ± 0.42 ^b^	3.76 ± 0.41 ^b^	3.14 ± 0.36 ^b^	0.252	0.0032
Alanine	2.03 ± 0.26 ^a^	1.45 ± 0.22 ^b^	1.40 ± 0.22 ^b^	1.11 ± 0.18 ^b^	0.129	0.0062
Arginine	2.17 ± 0.28 ^a^	1.40 ± 0.22 ^b^	1.35 ± 0.22 ^b^	1.21 ± 0.19 ^b^	0.132	0.0035
Cystine + cysteine	0.305 ± 0.049 ^a^	0.234 ± 0.038 ^ab^	0.207 ± 0.034 ^b^	0.167 ± 0.027 ^b^	0.022	0.0125
Phenylalanine	1.38 ± 0.22 ^a^	0.97 ± 0.16 ^b^	0.99 ± 0.16 ^b^	0.82 ± 0.13 ^b^	0.097	0.0181
Glycine	1.79 ± 0.25 ^a^	1.17 ± 0.19 ^b^	1.14 ± 0.18 ^b^	0.91 ± 0.15 ^b^	0.114	0.0034
Hydroxyproline	sub-0.010	sub-0.010	sub-0.010	sub-0.010	-	-
Isoleucine	1.60 ± 0.23 ^a^	1.22 ± 0.20 ^b^	1.16 ± 0.19 ^b^	0.94 ± 0.15 ^b^	0.112	0.0192
Histidine	1.10 ± 0.18 ^a^	0.80 ± 0.13 ^b^	0.78 ± 0.13 ^b^	0.68 ± 0.11 ^b^	0.081	0.0290
Leucine	2.75 ± 0.32 ^a^	2.06 ± 0.27 ^b^	1.95 ± 0.26 ^b^	1.59 ± 0.23 ^b^	0.157	0.0053
Lysine	3.19 ± 0.36 ^a^	2.30 ± 0.29 ^b^	2.14 ± 0.27 ^bc^	1.72 ± 0.24 ^c^	0.169	0.0020
Methionine	0.82 ± 0.14 ^a^	0.585 ± 0.099 ^b^	0.535 ± 0.091 ^b^	0.418 ± 0.067 ^b^	0.059	0.0080
Proline	1.36 ± 0.22 ^a^	1.07 ± 0.17 ^ab^	1.07 ± 0.18 ^ab^	0.88 ± 0.15 ^b^	0.106	0.072
Serine	1.37 ± 0.22 ^a^	1.01 ± 0.16 ^b^	0.95 ± 0.15 ^b^	0.80 ± 0.13 ^b^	0.098	0.019
Tyrosine	1.09 ± 0.18 ^a^	0.70 ± 0.11 ^b^	0.70 ± 0.12 ^b^	0.625 ± 0.100 ^b^	0.076	0.009
Threonine	1.62 ± 0.24 ^a^	1.25 ± 0.20 ^ab^	1.15 ± 0.19 ^b^	0.88 ± 0.15 ^b^	0.115	0.012
Valin	1.63 ± 0.24 ^a^	1.20 ± 0.20 ^b^	1.15 ± 0.19 ^b^	0.94 ± 0.15 ^b^	0.114	0.016
Total amino acids	32.911 ± 1.126 ^a^	23.966 ± 0.891 ^b^	22.929 ± 0.865 ^b^	18.872 ± 0.739 ^c^	0.529	0.000

SEM—common standard error of the means; *p*-value—global test (ANOVA) for the comparison between the 4 batches for the same parameter; M—control sample; G1—sample with 5% WB; G2—sample with 10% WB; and G3—sample with 15% WB. Values within the same row bearing distinct letters denote statistically significant variations (*p* < 0.05).

**Table 7:** Wheat bran effect on the antioxidant activity of meatballs expressed as DPPH radical scavenging ability (%).

Storage, Days	DPPH-RSA				SEM	*p*-Value ANOVA
	M	G1	G2	G3		
0	70.11 ± 0.10 ^d^	77.72 ± 0.89 ^c^	80.31 ± 0.51 ^b^	84.73 ± 0.54 ^a^	0.335	<0.001
3	86.89 ± 0.10 ^a^	75.13 ± 1.30 ^d^	79.86 ± 1.48 ^c^	84.11 ± 0.31 ^b^	0.575	<0.001
6	82.80 ± 0.11 ^a^	71.63 ± 0.38 ^b^	70.83 ± 0.91 ^b^	83.29 ± 0.56 ^a^	0.329	<0.001
9	68.45 ± 1.26 ^a^	58.17 ± 1.24 ^c^	56.50 ± 0.62 ^c^	61.27 ± 0.37 ^b^	0.550	<0.001

SEM—common standard error of the means, for each day of storage; *p*-value—global test (ANOVA) for the comparison between the 4 batches on the same day; M—control sample; G1—sample with 5% WB; G2—sample with 10% WB; and G3—sample with 15% WB. Within the same day, values with distinct letters differ statistically significantly (*p* < 0.05).

**Table 8:** Instrumental texture profile analysis of the meatballs.

Parameter	Samples				SEM	*p*-Value ANOVA
	M	G1	G2	G3		
Hardness (N)	42.903 ± 1.627 ^a^	31.303 ± 1.732 ^b^	27.372 ± 1.763 ^c^	21.303 ± 1.156 ^d^	0.917	0.00000117
Cohesiveness	0.513 ± 0.038 ^a^	0.461 ± 0.019 ^b^	0.311 ± 0.017 ^c^	0.249 ± 0.016 ^d^	0.014	0.00000275
Gumminess (N)	22.337 ± 1.126 ^a^	19.641 ± 0.908 ^b^	16.433 ± 0.612 ^c^	13.134 ± 1.462 ^d^	0.620	0.00003241

SEM—common standard error of the means, for each day of storage; *p*-value—global test (ANOVA) for the comparison between the 4 batches on the same day; M—control sample; G1—sample with 5% WB; G2—sample with 10% WB; and G3—sample with 15% WB. Within the same day, values with distinct letters differ statistically significantly (*p* < 0.05).

## Data Availability

The original contributions presented in this study are included in the article. Further inquiries can be directed to the corresponding author.
